# Safety and efficacy of dendritic cell vaccine for COVID-19 prevention after 1-Year follow-up: phase I and II clinical trial final result

**DOI:** 10.3389/fimmu.2023.1122389

**Published:** 2023-06-19

**Authors:** Jonny Jonny, Terawan Agus Putranto, Martina Lily Yana, Enda Cindylosa Sitepu, Raoulian Irfon, Bunga Pinandhita Ramadhani, Muchlis Achsan Udji Sofro, Yetty Movieta Nency, Endang Sri Lestari, Ria Triwardhani, Mujahidah, Retty Karisma Sari, Nur Alaydrus Soetojo

**Affiliations:** ^1^ Installation of Cellcure Development, Gatot Soebroto Central Army Hospital, Jakarta, Indonesia; ^2^ Faculty of Medicine University of Pembangunan Nasional “Veteran” Jakarta, Jakarta, Indonesia; ^3^ Dr. Kariadi Hospital/Faculty of Medicine, Diponegoro University, Semarang, Indonesia

**Keywords:** COVID-19, vaccine, dendritic cells, SARS-CoV-2, clinical trial, autologous vaccines

## Abstract

**Introduction:**

Interim analysis of phase I and phase II clinical trials of personalized vaccines made from autologous monocyte-derived dendritic cells (DCs) incubated with S-protein of SARS-CoV-2 show that this vaccine is safe and well tolerated. Our previous report also indicates that this vaccine can induce specific T-cell and B cell responses against SARS-CoV-2. Herein, we report the final analysis after 1 year of follow-up regarding its safety and efficacy in subjects of phase I and phase II clinical trials.

**Methods:**

Adult subjects (>18 years old) were given autologous DCs derived from peripheral blood monocytes, which were incubated with the S-protein of SARS-CoV-2. The primary outcome is safety in phase I clinical trials. Meanwhile, optimal antigen dosage is determined in phase II clinical trials. Corona Virus Disease 2019 (COVID-19) and Non-COVID-19 adverse events (AEs) were observed for 1 year.

**Results:**

A total of 28 subjects in the phase I clinical trial were randomly assigned to nine groups based on antigen and Granulocyte-Macrophage Colony Stimulating Factor (GM-CSF) dosage. In the phase II clinical trial, 145 subjects were randomly grouped into three groups based on antigen dosage. During the 1-year follow-up period, 35.71% of subjects in phase I and 16.54% in phase II had non-COVID AEs. No subjects in phase I experienced moderate–severe COVID-19. Meanwhile, 4.31% of subjects in phase II had moderate–severe COVID-19. There is no difference in both COVID and non-COVID-19 AEs between groups.

**Conclusions:**

After 1 year of follow-up, this vaccine is proven safe and effective for preventing COVID-19. A phase III clinical trial involving more subjects should be conducted to establish its efficacy and see other possible side effects.

## 1 Introduction

Since being designated by the WHO as a pandemic in March 2019, SARS-CoV-2 has infected more than 540 million people and was responsible for 6 million deaths in June 2022 (1). In addition, this virus continues to mutate so that new variants emerge (2). Although the use of the SARS-CoV-2 vaccine currently can control COVID-19 infection and mortality, various studies, including meta-analyses, show a decrease in vaccine efficacy by up to 30% within 6 months after vaccination and a reduction in the ability of vaccines against emerging SARS-CoV-2 variants (3, 4). The problem is also exacerbated by the lack of optimal vaccination coverage caused by various factors and the public’s rejection of the current SARS-CoV-2 vaccine (5). Therefore, it is still necessary to develop vaccines that can last a long time, are effective against various variants, and increase vaccination coverage and public acceptance.

The development of dendritic cell (DC)–based vaccines is an innovative vaccine that can overcome existing problems. DC–based vaccines utilize the ability of DCs as antigen-presenting cells (APCs) to induce a human immune system oriented to T-cell immunity (6). The development of autologous DC–based vaccines with the *ex vivo* method can be an effective method because it can ensure the quality of the DCs used, streamline the DC maturation process and antigen presentation that occurs, and improve the safety of vaccination, including in subjects with comorbidities who have vaccination contraindications. In addition, autologous vaccines have the potential to increase public acceptance of vaccination (7).

In previous studies, both preclinical and interim analysis results of phase I and II clinical trials found that this vaccine has good potential. In short-term observations (3 months), no serious adverse event (SAE) was found in the subjects of phase I and II clinical trials. In addition, autologous DC–based vaccines loaded with SARS CoV-2 S-protein (AV-COVID-19 or Nusantara vaccine) can induce adequate T-cell immunity well. The vaccine can also form an antibody response (8). This article will present the safety results during 1-year observations. The efficacy potential of DC–based vaccines was also analyzed.

## 2 Methods

### 2.1 Study oversight and outcomes

The study was randomized and double-blinded phase 1 and 2 clinical trials. The phase I clinical trial was carried out at Dr. Kariadi Hospital Semarang, Faculty of Medicine, Diponegoro University, from December 2020 to December 2021. The phase II clinical trial was carried out at Gatot Subroto Hospital from April 2021 to May 2022. The research protocol follows the Helsinki declaration and has received ethical approval. Data security is reviewed by an independent Data Safety Monitoring Board (DSMB). The main objective of phase I clinical trials is to determine the safety of AV-COVID-19 vaccines, whereas phase II clinical trials aim to determine the optimal dose of SARS-CoV-2 antigens used in forming immunogenicity. Then, the safety and efficacy of the vaccine was also analyzed based on the immune response to the SARS CoV-2 protein. This study is registered in the US National Library of Medicine ClinicalTrials.gov under identifier NCT05007496. This research was sponsored by PT. Aivita Biomedika Indonesia.

### 2.2 Participants, randomization, and blinding

In phase I, as many as 106 prospective subjects were screened, and 33 subjects met the criteria and received the vaccine. Meanwhile, in phase II, as many as 227 prospective subjects were screened, and 145 subjects met the criteria and received the vaccine. The subjects included in this study were over 18 years old, generally healthy, not pregnant or planning to get pregnant for productive women, willing to comply with research protocols, and provided informed consent in writing. While the exclusion criteria consist of subjects with symptoms of active COVID-19 infection, positive PCR test results infected with SARS-CoV-2 less than 3 months, rapid examination of reactive SARS-CoV-2 IgG antibodies, having received other COVID-19 vaccines, positive pregnant, subjects with immunodeficiency diseases (HIV infection, Hepatitis C, and Hepatitis B), taking immunosuppressive drugs and corticosteroids for a long time, conditions that require oxygen supplementation, diagnosed with invasive cancer and receiving anticancer therapy, a history of thromboembolism/genetic predisposition of thromboembolic events/in antithromboembolic treatment other than low-dose aspirin, physical/mental disabilities that make a person unable to carry out daily activities, obesity (BMI > 40), uncontrolled hypertension (cystole > 180, diastole > 100), and unwilling to sign written consent. In phase I, in addition to the above conditions, subjects with uncontrolled, autoimmune laboratory abnormalities based on the Food and Drug Administration was excluded, as well as a history of obtaining blood products within 3 months.

All subjects that met the criteria were then randomized. In phase I, the subjects were divided into nine groups based on three antigen dose groups (0.1, 0.33, and 1.0 mcg) and three GM-CSF groups administered (0, 250, and 500 mcg). In phase II, subjects were divided into three different dose groups (0.1, 0.33, and 1.0 mcg). Neither the researchers nor the subjects knew the doses of antigens and GM-CSF.

### 2.3 Vaccine

The vaccine consists of DCs and lymphocytes obtained from the blood of the peripheral autologous. First, 40 cc of blood was taken and then incubated with GM-CSF and IL-4 for 5 days to differentiate monocytes into DCs. Then, DCs and lymphocytes were incubated with recombinant SARS-CoV-2 S protein for 2 days. S protein is manufactured by LakePharma Biological (San Carlos, USA). The antigen is SARS-CoV-2 spike (S) protein pre-fusion stabilized ectodomain, C-terminus His tag, with the furin cleavage site removed and trimerization stabilized. The sequence is based on SARS-CoV-2 wild type. The amount of S protein given differs according to the dose group, namely, 0.1, 0.33, and 1.0 mcg. The vaccine composition consists of 10% DCs and 90% lymphocytes. In phase I clinical trials, a DC vaccine was given an additional GM-CSF of 250 or 500 mcg, while in phase II clinical trials, it was not given additionally. The formed DC vaccine is dissolved in the subject’s plasma before injection. Vaccines are produced following the requirements of a Good Manufacturing Product.

### 2.4 Procedure

Screening of all potential subjects by medical staff was carried out. In the phase I clinical trial, eligible subjects were randomly divided into nine groups based on dose and GM-CSF addition, while in the phase II clinical trial, the subjects were divided into three groups. Then, 40 cc peripheral blood were drawn from subjects, which was processed into a DC vaccine. Seven days after the blood draw, the subject came back. If there are no signs of being infected with COVID-19, 6 cc of blood is taken again to evaluate baseline conditions before vaccination for hematology, BUN, creatinine, sodium, potassium, chloride, phosphorus, calcium, lipid profile, SGOT, SGPT, total protein, albumin, globulin, alkaline phosphatase, total bilirubin, direct bilirubin, and lipase. In patients with diabetes mellitus, fasting blood sugar and HbA1c tests are carried out, while in non-diabetic patients, only blood sugar is carried out. The vaccine was injected subcutaneously, and subjects were monitored for 30 min to assess any unintended reactions or events after vaccine administration. Then, the subjects were asked to report the local and systemic reactions they experienced within 7 days post-vaccination using a recall card. Safety evaluations were conducted again on the 7th- and 28th-day visits. The subject was then monitored again in the 3rd, 6th, 9th, and 12th months for monitoring of unwanted events. In phase II, an Enzyme-linked immunosorbent spot (ELISPOT) examination is carried out at week 2 and 4 to determine the immunogenicity of the vaccine. All data are stored in a manual and electronic Case Report Form (CRF).

### 2.5 Safety assessment

Safety assessments are carried out by evaluating local and systemic reactions experienced by post-vaccination subjects. Subjects were monitored for 30 min post-vaccination to assess the immediate effects of the vaccine. On the 7th and 28th days after vaccination, we conducted a safety laboratory examination consisting of haematology, blood urea nitrogen (BUN), creatinine, sodium, potassium, chloride, phosphorus, calcium, lipid profile, SGOT, SGPT, total protein, albumin, globulin, alkaline phosphatase, total bilirubin, direct bilirubin and lipase. Fasting blood glucose were examined in subjects with diabetes mellitus, meanwhile non-fasting blood glucose were examined in non-diabetic subjects. Patients are continuously monitored to assess unwanted events that occurred in the 3rd, 6th, 9th, and 12th months after vaccination.

### 2.6 Immunogenicity and effective dose assessment

In phase II, the determination of an effective dose is assessed based on the immunogenicity of the vaccine. The doses of the SARS-CoV-2 S protein composition tested were 0.10, 0.33, and 1.00 mcg. The immune response to detect specific memory T cells against SARS-CoV-2 was assessed by performing an ELISPOT examination measuring interferon-gamma.

### 2.7 Efficacy assessment

The efficacy assessment was assessed by looking at the immunogenicity of the vaccine through the results of the ELISPOT examination at weeks 2 and 4. In addition, clinical trial subjects were followed for up to 1 year and incidence rates of COVID-19 that occurred were recorded. Efficacy is assessed by the vaccine’s protective ability to prevent hospitalization due to COVID-19.

### 2.8 Statistical analysis

In phase, I and phase II, vaccine safety and efficacy analysis for up to 1 year were performed on all vaccinated subjects (intention-to-treat). Vaccine safety is presented descriptively by showing the proportion of unintended events in each dose group. The efficacy of the vaccine for up to 1 year was presented descriptively by showing the proportion of COVID-19 incidence in each dose group. If you meet the requirements (the number of expected counts <5 does not exceed 20%), then the difference in proportion is tested using chi-square. However, if it does not meet the requirements, the proportion difference test is carried out using Fisher’s exact test.

## 3 Result

### 3.1 Subject characteristics

In the phase, I clinical trial, a total of 28 subjects received the vaccine and were grouped into nine groups. A total of 16 subjects (57.14%) were men, and 12 (42.83%) were women. The subjects of the study were in the age range of 18–61 years for men and 23–60 years for women. In the phase II clinical trial, a total of 145 subjects received the vaccine and were grouped into three groups (**Table 1**). In phase II clinical trials, there were several comorbidities in subjects such as hypertension, diabetes mellitus, and asthma. Meanwhile, in phase I clinical trials, these comorbidities were part of exclusion criteria. There were no significant differences in characteristics between the trial groups in either phase I or II clinical trials.

**Table 1:** Demographic characteristics of phase II clinical trial subjects.

Characteristic	0.10 mcg (n = 49)	0.33 mcg (n = 49)	1.00 mcg (n = 47)	Total (n = 145)
**Mean Age in years ± SD**	45.3 ± 13.3	47.5 ± 12.3	44.9 + 12.4	45 ±13
Sex				
** Male**	27 (55.1%);	22 (45.9%);	28 (59.6%);	77 (53.1%);
** Female**	22 (45.9%)	27 (55.1%)	19 (40.4%)	68 (46.9%)
Race*/*Ethnicity				
** Javanese**	21 (42.9%);	16 (32.7%);	23 (48.9%);	60 (41.4%);
** Tionghoa**	13 (26.5%);	18 (36.7%);	12 (25.5%);	43 (29.7%);
** Sundanese**	4 (8.2%);	4 (8.2%);	3 (6.4%);	11 (7.6%);
** Minangkabau**	3 (6.1%);	2 (4.1%);	3 (6.4%);	8 (5.5%);
** Bataknese**	1 (2.0%);	3 (6.1%);	1 (2.2%);	5 (3.4%);
** Sumatran**	2 (4.1%);	1 (2.0%);	0 (0%);	3 (2.1%);
** Malaysia**	1 (2.0%);	0 (0%);	2 (4.3%);	3 (2.1%);
** Buginese**	1 (2.0%)	1 (2.0%)	1 (2.2%)	3 (2.1%)
Comorbidities				
** Gastric reflux**	0 (0%)	2 (4.1%)	1 (2.2%)	3 (2.1%)
** Hypertension**	6 (12.2%);	5 (10.2%);	4 (8.5%);	15 (10.3%);
** Hypercholesterolemia**	1 (2.0%);	1 (2.0%);	2 (4.3%);	4 (2.8%);
** Diabetes**	1 (2.0%);	1 (2.0%);	8 (17%);	10 (6.9%)
** Asthma**	3 (6.1%);	3 (6.1%);	1 (2.2%);	7 (4.8%);
** Hyperuricemia/Gout**	0 (0%);	2 (4.1%);	3 (2.1%);	3 (2.1%);

### 3.2 Safety

AEs occurring up to 28 days post-vaccination in phase I and phase II clinical trial subjects have been previously reported where there were no acute allergic reactions, SAE, or AE of degree 3 or 4[8]. In phase, I, subjects who experienced AE degrees 1 and 2 to 28 days were 64.5%. Meanwhile, in phase II, which experienced AE degrees 1 and 2, it was 53.6%. The incidence of AE did not differ significantly between the three antigen dose groups, except that the proportion without AE was significantly higher in the 0.10 μg group when compared to the 1.00 μg group.

Observations of the presence of AE occurred were made on all subjects of phase I and phase II clinical trials for up to 1 year. In addition to the incidence of COVID-19, 10 AEs that happened were not related to vaccine administration in phase I clinical trial subjects (**Table 2**). Meanwhile in the phase II clinical trial, a total of 25 AEs not related to vaccine administration occurred. One of these incidents classified as an SAE is retinal detachment, which requires surgery but is not related to the vaccine administered. There was no significant difference (p = 0.664) in the proportion of AE among the antigen dose groups (**Table 3**). The most AE occurred in the 1.0 μg antigen dose group.

**Table 2:** Adverse event (AE) phase I clinical trials during a follow-up period of 1–12 months post-vaccination.

Time	Adverse Event	Non GM-CSF (n = 9)	GM-CSF 250 mcg (n = 9)	GM-CSF 500 mcg (n = 10)
Month-3	Local reaction	0 (0%)	0 (0%)	0 (0%)
	Systemic reaction	2 (22.2%)	3 (33.3%)	1 (10%)
Month-6	Local reaction	0 (0%)	0 (0%)	0(0%)
	Systemic reaction	1 (11.1%)	1 (11.1%)	1 (10%)
Month-9	Local reaction	0 (0%)	0 (0%)	0 (0%)
	Systemic reaction	1 (11.1%)	0 (0%)	0 (0%)
Month-12	Local reaction	0 (0%)	0 (0%)	0 (0%)
	Systemic reaction	0 (0%)	0 (0%)	0 (0%)
	Adverse Event	Antigen Dose 0.10 mcg (n = 9)	Antigen Dose 0.33 mcg (n = 9)	Antigen Dose 1.00 mcg (n = 10)
Month-3	Local reaction	0 (0%)	0 (0%)	0 (0%)
	Systemic reaction	3 (33.3%)	2 (22.2%)	1 (10%)
Month-6	Local reaction	0 (0%)	0 (0%)	0 (0%)
	Systemic reaction	1 (11.1%)	1 (11.1%)	1 (10%)
Month-9	Local reaction	0 (0%)	0 (0%)	0 (0%)
	Systemic reaction	1 (11.1%)	0 (0%)	0 (0%)
Month-12	Local reaction	0 (0%)	0 (0%)	0 (0%)
	Systemic reaction	0 (0%)	0 (0%)	0 (0%)
Total	Local reaction	0	0	0
	Systemic reaction	5 (55.5%)	3 (33.3%)	2 (20%)

**Table 3:** AE phase II clinical trials during a follow-up period of 1–12 months post-vaccination.

Adverse Event	0.1 mcg (N = 48)	0.33 mcg (N = 47)	1.00 mcg (N = 43)
Non-COVID respiratory infection	3 (6.25%)	6 (12.76%)	7 (16.28%)
Hypertension	1 (2.08%)	0 (0%)	1 (2.32%)
Type II diabetes mellitus	0 (0%)	0 (0%)	1 (2.32%)
Coronary artery disease	0 (0%)	1 (2.13%)	0 (0%)
Fatty liver	1 (2.08%)	0 (0%)	0 (0%)
Unspecified infection	1 (2.08%)	0 (0%)	0 (0%)
Retinal detachment	1 (2.08%)	0 (0%)	0 (0%)
**Total (p = 0.664)**	**7 (14.58%)**	**7 (14.89%)**	**9 (20.9%)**

The meaning of bold values are the result from our study.

### 3.3 Efficacy

Evaluation of the ELISPOT assay test shows that 38.8% of subjects were reactive to SARS-CoV-2 S protein (significant increase of spot count) at day 14. This number increases where 62.2% of subjects were reactive to SARS-CoV-2 S protein at day 14 or day 28 (**Table 4**). There is no significant difference of proportion between the dose group.

**Table 4:** SARS-CoV-2-specific T-cell memory response.

	Formulation by the quantity of S-protein incubated with DC			
	0.01 mcg	0.33 mcg	0.1 mcg	Total
Proportion reactive by antigen-stimulated ELISPOT assay at day-14*	14/33 (42.4%)	13/31 (41.9%)	11/34 (32.4%)	38/98 (38.8%)
Proportion reactive by antigen-stimulated ELISPOT assay test at day-14 or day-28*	22/33 (66.7%)	21/31 (67.7%)	18/34 (52.9%)	61/98 (62.2%)

^*^ELISPOT was conducted using venous peripheral blood, reactivity defined as statistically significant increase of spot count on in vitro antigen-stimulated (SARS-CoV-2 S protein) ELISPOT compared to unstimulated ELISPOT. There was no difference in any 2 × 2 comparison of proportions with increased ELISPOTS based on quantity of S-protein used in manufacturing (p > 0.05, X^2^).

**Figure 1:**
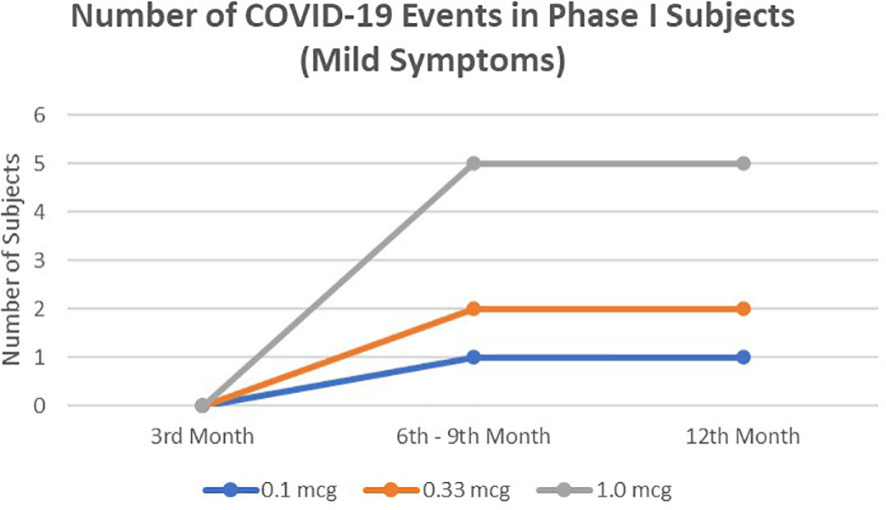
The number of mild symptomatic COVID-19 events from 3rd to 12thmonths in phase I subjects. No severe symptomatic COVID-19 events were observed.

**Figure 2:**
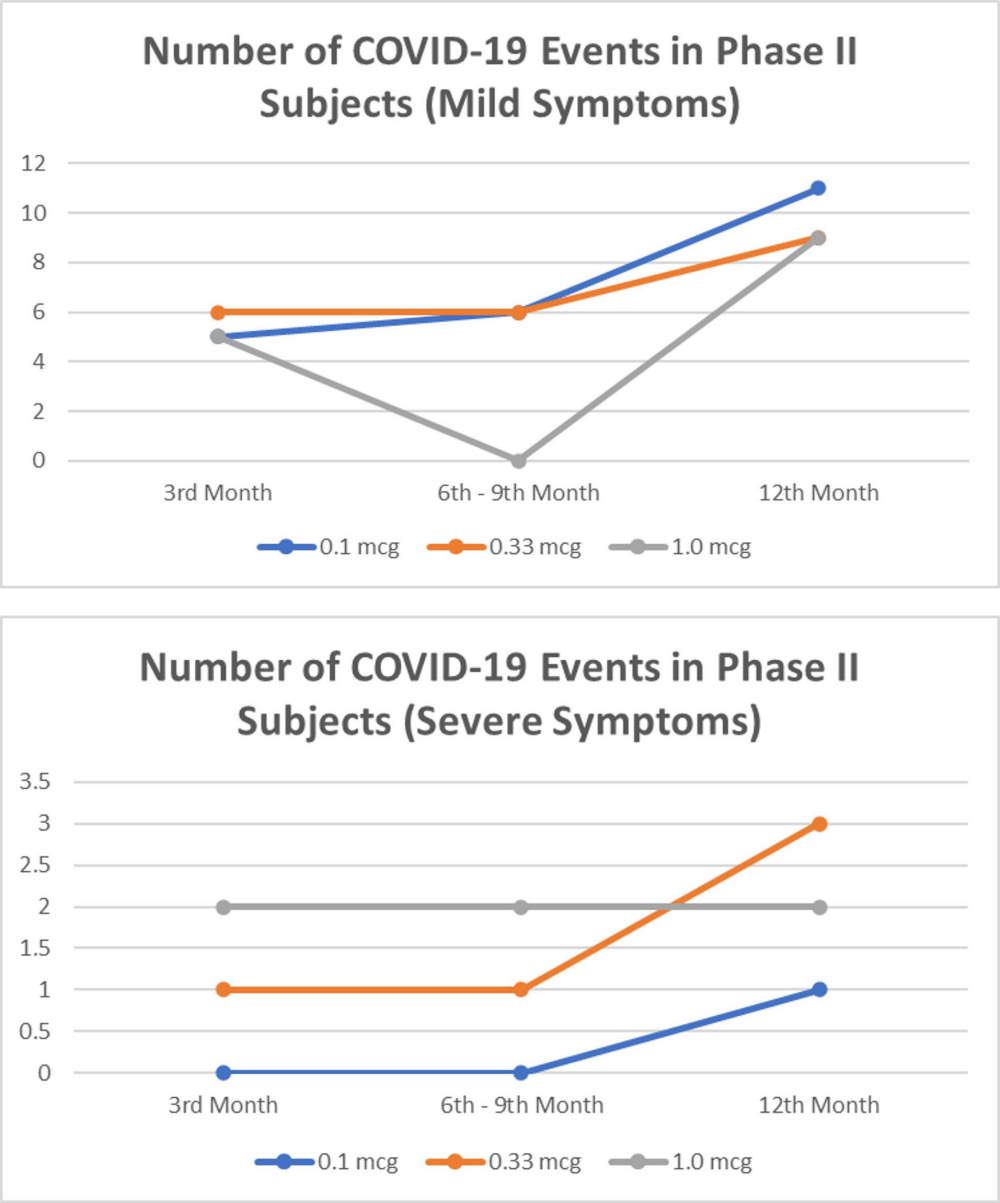
The number of mild and severe symptomatic COVID-19 events from 3rd to 12th months in phase II subjects. No significant difference between dose group (p>0.05).

In a 1-year observation of the subjects of the phase I clinical trial, eight people were confirmed positive for SARS-CoV-2 infection, all of whom were mild clinical symptoms and did not require hospital treatment (**Figure 1**). Meanwhile, in a phase II clinical trial in 1-year observation, 36 subjects were confirmed positive for SARS-CoV-2 infection (**Figure 2**). Of the 36 subjects, one subject died as a result of severe symptomatic SARS-CoV-2 infection. Overall in the phase II clinical trial, the incidence of SARS-CoV-2 infection was 30 people infected with mild degrees and received self-care at home, while severe degrees and requiring hospitalization were six subjects consisting of four subjects in the usual care ward and two subjects requiring ICU treatment.

## 4 Discussion

### 4.1 Safety

AEs that occur up to 28 days post-vaccination, based on reported data, are all mild. Non-COVID AE for up to 28 days was reported by 64.5% of phase I subjects and 53.6% of phase II subjects, and there were no significant differences between treatment groups (8). Similarly, in follow-ups from 1 to 12 months, the total incidence of non-COVID AE in phase I was 35.71% of subjects. Meanwhile, in phase II, the total incidence of non-COVID AE was 16.67% of subjects. From these data, it can be concluded that this vaccine is safe. Even with AEs at 1–12 months, the incidence rate is lower than 28 days post-vaccination, both in phase 1 and phase 2.

The short-term AE incidence rate of DC vaccines is lower compared to conventional COVID-19 vaccines (8). The grade 3 non-COVID AEs for up to 1 year found in other reported COVID-19 vaccine studies was 1.6% (**Table 5**) (13). Several other COVID-19 vaccines that have been widely circulated to date have only reported COVID-19 related AE that occurs for up to 4–9 months and based on the report, AE grade 3 that occurs in other COVID-19 vaccines ranges from 0.1%–1.6% (**Table 5**) (9–12, 14). Meanwhile, in this study, only one case of AE grade 3 (0.72%) was found. Thus, the AE found in this study was lower than other COVID-19 vaccines. A low AE in DC–based vaccines was also found in previous DC vaccine studies (15–17). After two decades of observations on various phase I and II clinical trials, the DC vaccine was well tolerated and induced minimal toxicity. The most frequent manifestations of AEs are local reactions at the injection site, such as rashes and pruritus, and sometimes, systemic effects include fever and malaise (18). The low AE in this vaccine is caused by the autologous formulation of the vaccine so that there is no exposure to foreign bodies that can trigger excessive inflammatory reactions.

**Table 5:** AE Non-COVID up to 1 year COVID-19 vaccine clinical trial.

Study	Number of Subjects	Overall AE Non-Covid	AE Grade ≥3	Follow-Up Period
**AV-COVID-19**	**138 (phase II)**	**16,67%**	**0.72%**	**365 days**
Astrazeneca ChAdOx1 nCoV-19 (9)	17.662 (phase III)	NA	0.4%	135 days
J&J Ad26.COV2.S (4)	19.113 (phase III)	NA	1%	294 days
Moderna mRNA-1273 (10)	14.287 (phase III)	NA	1.3%	260 days
Pfizer BNT162b2 (11)	21.650 (phase III)	NA	1.2%	238 days
Sinovac CoronaVac (12)	6.646 (phase III)	NA	0.1%	120 days
Zififax ZF2001 (13)	12.625 (phase III)	NA	1.6%	390 days

NA, Data not available.

* Reported data are AE phase III clinical trials in the treatment group.

The meaning of bold values are the result from our study.

There was an incidence of type 2 diabetes mellitus, hypertension, coronary artery disease, fatty liver, retinal detachment, and other infections in phase II subjects (**Table 3**). However, there was no significant difference in the proportion of incidence of these diseases among the dose group. In addition to the incidence of COVID-19, there was reportedly only one severe AE event, retinal detachment, which occurred in the 0.1 mcg antigen formulation group. Compared to other COVID-19 vaccines, vaccination is associated with an increased risk of some diseases. In the phase III clinical trial of an adenovirus vector–based COVID-19 vaccine, more incidence of tinnitus, urticaria, pulmonary embolism, and deep-vein thrombosis was found in subjects who received the vaccine than placebo (14). This is in line with findings in mRNA-based COVID-19 vaccines, which are linked to the incidence of myocarditis, thrombocytopenia, sexual dysfunction, and hypercoagulation (19–21). No incidence of these diseases was found in phase 1 and 2 clinical trials of this vaccine. The causes of the emergence of those diseases after the COVID-19 vaccination are still under study. One of the hypotheses that emerged is molecular mimicry, where viral antigens given to conventional vaccines resemble self-proteins, triggering cross-reactivity in susceptible individuals (21). In the DC vaccine, antigens are introduced to DC *ex vivo* so that the risk of cross-reactivity with self-protein in the body can be avoided. Although the risk of certain diseases arising from the COVID-19 vaccine is relatively low, the widespread and long-term use of the vaccine that causes these incidents should be monitored and considered in future studies.

### 4.2 Efficacy and antigen dosage

ELISPOT results show that this vaccine can induce SARS-CoV-2-specific memory T cells (**Table 4**). The number of reactive result increases from 38.8% to 62.2% on day 28. There is no difference of proportion between the dose group so that 0.01 mcg are adequate to induce cellular immunity response. Presentation of antigens to T cells by DCs is a highly efficient process (22) so that a small amount of antigens is capable to induce T-cell responses. The lowest dose of antigens in this study is the most optimal dose.

Although phase II aims to determine the optimal dose of antigens, in this study, we also evaluated the efficacy of this vaccine in preventing the onset of moderately and severely symptomatic COVID-19 that requires hospital treatment. From a follow-up for 1 year, six subjects experienced moderate–severe symptomatic COVID-19 infection that required hospital treatment in phase II subjects. Thus, it was found that this vaccine protected 95.56% (intention-to-treat analysis, N = 138) of subjects from hospitalization due to COVID-19 for up to 1 year. When compared between dose groups, the proportion of subjects infected with COVID-19, both mild degrees undergoing self-isolation and severe degrees requiring hospital treatment, did not differ significantly. Therefore, there was no difference in efficacy between dose groups in preventing COVID-19 infection and preventing hospitalization. Based on its safety profile and immunogenicity for up to 28 days, the 0.1 mcg antigen dose group was designated as the optimal dose. In the evaluation for up to 1 year, there was no significant difference between the antigen dose groups in preventing the onset of moderately and severely symptomatic COVID-19, thus further supporting the selection of a dose of 0.1 mcg as the most optimal antigen dose formulation.

Clinical trials of conventional COVID-19 vaccines mostly use humoral responses to assess vaccine immunogenicity, although correlates of protection from COVID-19 vaccines have not yet been established (23). However, there is growing evidence that the T-cell response plays a significant role in the elimination of the COVID-19 virus and is thought to play a central role in forming broad-spectrum and long-term immunity (24–26). In this clinical trial, monitoring for up to 28 days showed that vaccine administration could trigger an optimal specific T-cell response to SARS-CoV-2 (8). Data from phase I also show good induction of humoral responses. At the same time, the response rate of T cells is not different in all formulation groups. The consistent rate of efficacy for up to 1 year proves that the specific T-cell immune response to SARS-CoV-2 formed from administering the DC vaccine can last a long time. Therefore, it is quite possible that booster vaccine might not be necessary.

Compared to the results of clinical trials of conventional vaccines, the proportion of COVID-19 AE incidence up to 1 year in this vaccine tends to be higher. However, this is likely because this study is a phase II clinical trial with a small number of subjects (**Table 6**). In addition, this study was conducted in Indonesia. During the wide spread of SARS-CoV-2, omicron and delta variants were more infectious and caused more severe symptoms (27, 28). Therefore, a much larger number of samples is needed to determine their effectiveness in preventing COVID-19 infection in the population. Therefore, it is necessary to conduct phase III clinical trials with a more significant number of subjects so that the efficacy of this vaccine in preventing COVID-19 infection in the population can be established.

**Table 6:** AE COVID-19 up to 1 year COVID-19 vaccine clinical trial.

Study	Number of Subjects	Overall Symptomatic COVID-19	COVID-19 Grade ≥3	Follow-Up Period
**AV-COVID-19**	**139 (phase II)**	**36 (25,89%)**	**6 (4.31%)**	**365 days**
Astrazeneca ChAdOx1 nCoV-19 (9)	17.662 (phase III)	0.4%	<0.1%	135 days
J&J Ad26.COV2.S (4)	19.113 (phase III)	2.31%	2.27%	294 days
Moderna mRNA-1273 (10)	14.287 (phase III)	0.38%	0.75%	260 days
Pfizer BNT162b2 (11)	21.650 (phase III)	0.37%	0.005%	238 days
Sinovac CoronaVac (12)	6.646 (phase III)	1.11%	0%	120 days
Zififax ZF2001 (13)	12.625 (phase III)	1.25%	0.047%	390 days

The meaning of bold values are the result from our study.

### 4.3 Feasibility

This research shows that personalized DC–based vaccines can be manufactured in pandemic situations. This clinical trial was conducted in two hospital centers with a production time of 1 week. Trained staff can produce vaccines in health facilities/hospitals with specific standard preparations. This vaccine is unsuitable for the mass vaccination program, which requires fast production time. However, due to its safety and ‘personal’ source, this vaccine is a promising option for subjects who cannot meet conventional vaccine criteria for medical reasons (e.g., in autoimmune and cancer patients) or non-medical (rejection religion/belief), and this needs to be continued with the phase III clinical trial with a larger number of subjects. Furthermore, this study used S-proteins that proved effective. This can cut the exploration time of antigen selection. However, it is necessary to consider using other antigens, for example, by adding variant antigens or designing proteins conserved in variants. This vaccine can be one of the COVID-19 vaccine options, thus expanding the range of COVID-19 vaccination.

## 5 Conclusion

Based on phase I and II clinical trials of DC–based COVID-19 prevention vaccines, the absence of SAE associated with vaccine administration for up to 1 year for SARS-CoV-2 infection shows good tolerability and safety. Of the three vaccine dose candidates tested, the smallest dose of 0.1 mcg provides good immunogenicity and is supported by minimal AE. A 1-year evaluation shows that this vaccine appears to have long-term immunogenic potential. The phase III trial with a larger number of subjects is needed to ensure the efficacy of this vaccine and see other possible side effects.

## Data availability statement

The raw data supporting the conclusions of this article will be made available by the authors, without undue reservation.

## Ethics statement

The studies involving human participants were reviewed and approved by Gatot Soebroto Army Hospital. The patients/participants provided their written informed consent to participate in this study.

## Author contributions

TP, YN, EL, RT, M, RS, and NS contributed to the design, implementation, and analysis of the phase I trial. JJ, TP, MY, ES, RI, and BR contributed to the design, implementation, and analysis of the phase II trial. JJ, ES, RI, ES, and YN wrote the manuscript. All authors contributed to the article and approved the submitted version.
